# Multiple imputation of missing covariates with non-linear effects and interactions: an evaluation of statistical methods

**DOI:** 10.1186/1471-2288-12-46

**Published:** 2012-04-10

**Authors:** Shaun R Seaman, Jonathan W Bartlett, Ian R White

**Affiliations:** 1 MRC Biostatistics Unit, Institute of Public Health, Forvie Site, Robinson Way, Cambridge CB2 0SR, UK; 2 Department of Medical Statistics, London School of Hygiene and Tropical Medicine, Keppel Street, London WC1E 7HT, UK

## Abstract

**Background:**

Multiple imputation is often used for missing data. When a model contains as covariates more than one function of a variable, it is not obvious how best to impute missing values in these covariates. Consider a regression with outcome *Y *and covariates *X *and *X*^2^. In 'passive imputation' a value X* is imputed for *X *and then *X*^2 ^is imputed as (X*)^2^. A recent proposal is to treat *X*^2 ^as 'just another variable' (JAV) and impute *X *and *X*^2 ^under multivariate normality.

**Methods:**

We use simulation to investigate the performance of three methods that can easily be implemented in standard software: 1) linear regression of *X *on *Y *to impute *X *then passive imputation of *X*^2^; 2) the same regression but with predictive mean matching (PMM); and 3) JAV. We also investigate the performance of analogous methods when the analysis involves an interaction, and study the theoretical properties of JAV. The application of the methods when complete or incomplete confounders are also present is illustrated using data from the EPIC Study.

**Results:**

JAV gives consistent estimation when the analysis is linear regression with a quadratic or interaction term and *X *is missing completely at random. When *X *is missing at random, JAV may be biased, but this bias is generally less than for passive imputation and PMM. Coverage for JAV was usually good when bias was small. However, in some scenarios with a more pronounced quadratic effect, bias was large and coverage poor. When the analysis was logistic regression, JAV's performance was sometimes very poor. PMM generally improved on passive imputation, in terms of bias and coverage, but did not eliminate the bias.

**Conclusions:**

Given the current state of available software, JAV is the best of a set of imperfect imputation methods for linear regression with a quadratic or interaction effect, but should not be used for logistic regression.

## Background

In most medical and epidemiological studies some of the data that should have been collected are missing. This presents problems for the analysis of such data. One approach is to restrict the analysis to complete cases, i.e. those subjects for whom none of the variables in the analysis model are missing. Data are said to be missing completely at random (MCAR), missing at random (MAR) or missing not at random (MNAR) [1]. MCAR means that that the probability of the pattern of missing data being as it is depends on neither the observed nor the missing data. MAR is the weaker condition that the probability does not depend on the missing data given the observed data. MNAR means that it depends also on the missing data. When data are MCAR, the complete cases constitute a representative subsample of the sample, and so the complete-case analysis is valid. However, when data are MAR, using only complete cases can yield biased parameter estimators. Furthermore, even when data are MCAR, this approach is inefficient, as it ignores information from incomplete cases.

A method for handling missing data that gives valid inference under MAR and which is more efficient than just using complete cases is multiple imputation (MI) [1]. Here a Bayesian model with non-informative prior is specified for the joint distribution of the variables in the analysis model, as well as possibly other ('auxiliary') variables. This model is fitted to the observed data assuming that they are MAR. A single imputed dataset is now created by sampling the parameters of the imputation model from their posterior distribution, in order to account for the uncertainty in this model, and then randomly generating ('imputing') values for the missing data using these sampled parameter values in the specified model. This procedure is repeated multiple times, so generating multiple imputed datasets, and then the analysis model is fitted to each of these in turn. Finally, the complete-data parameter and variance estimates from each imputed dataset are combined according to simple formulae called Rubin's Rules. Note that when some of the variables are fully observed, it is unnecessary to model their distribution and the imputation model can be a model for the conditional distribution of the remaining variables given these.

This article is concerned with the use of MI when the analysis model includes as covariates more than one function of the same variable and this variable can be missing. Such situations arise when the analysis model includes both linear and higher-order terms of the same variable or when the model includes an interaction term. This is the case, for example, when non-linear associations are explored using fractional polynomials or splines [2]. In such situations, the imputation is complicated by the functional relationship between the covariates in the analysis model. In this article we focus on two particular simple settings: where the analysis model is 1) linear regression of an outcome *Y *on covariates *X *and *X*^2^, and 2) linear regression of *Y *on covariates *X, Z *and *XZ*. These are the two settings considered by Von Hippel (2009) [3], who also investigated methods for imputing variables in the presence of higher-order or interaction effects. Unless stated otherwise, we suppose that *Y *and *Z *are fully observed and *X *can be missing. We investigate three methods of MI that can be easily implemented in standard software.

In 'passive imputation', an imputation model is specified for the distribution of *X *given *Y *(or *X *given *Y *and *Z*). Missing values of *X *are imputed from this model and the corresponding values of the function(s) (*X*^2 ^or *XZ*) of *X *calculated. Von Hippel (2009) [3] called this method 'impute then transform'. In principle, there is nothing wrong with this method. However, in practice, the existence of the higher-order or interaction effects makes commonly used imputation models misspecified. The conditional distribution of *X *given *Y *(and *Z*) depends on the distribution of *X *(and *Z*) and the conditional distribution of *Y *given *X *(and *Z*). In the case of a linear regression analysis model, if *X *(and *Z*) are (jointly) normally distributed and the true coefficient of the higher-order or interaction term in the analysis model is zero, the conditional distribution of X given *Y *(and *Z*) is given by the linear regression of *X *on *Y *(and *Z*). If the coefficient is not zero, this is no longer so. Nevertheless, such a linear regression model would commonly be used in practice as an imputation model for *X*.

It is possible that passive imputation might be improved by using predictive mean matching (PMM) [4]. In this approach, rather than using the imputation model to generate missing *X *values directly, it is used to match subjects who have missing *X *with subjects with observed *X*. Each incomplete case's missing *X *is then imputed as the matching subject's value of *X*. The motivation for PMM is that it may be more robust to misspecification of the imputation model and that grossly unrealistic imputed values are avoided, since every imputed value has actually been realised at least once in the dataset.

Passive imputation and PMM ensure that the imputed values conform to the known functional relation between the covariates, e.g. that the imputed value of *X*^2 ^is equal to the square of the imputed value of *X*. The third method of MI that we examine was recently proposed by Von Hippel (2009) [3]. This ignores the functional relation between covariates and treats a higher-order or interaction term as just another variable. Von Hippel called this approach 'transform then impute'; following White et al. (2011) [5], we call it JAV ('Just Another Variable'). In this method, missing *X *and *X*^2 ^(or *XZ*) are imputed under the assumption that *Y, X *and *X*^2 ^(or *Y, X, Z *and *XZ*) are jointly normally distributed. Corresponding imputed values of *X *and *X*^2 ^will not, in general, be consistent with one another, e.g. *X *may be imputed as 2 while *X*^2 ^is imputed as 5. However, Von Hippel argued that this does not matter for estimation of the parameters of the analysis model. We shall examine Von Hippel's argument in detail in the Results section.

In the present article we investigate, using simulation, the performance of three methods easily implemented in standard software -- passive imputation, PMM and JAV -- in the two settings described above. We look at bias of parameter estimators and coverage of confidence intervals. In addition to considering linear regression analysis models, we also look at the logistic regression of binary *Y *on *X *and *X*^2^. Von Hippel justified the use of JAV for a linear regression analysis model, but suggested that it might also work well in the setting of logistic regression, because the logistic link function is fairly linear except in regions where the fitted probability is near to zero and one. In the Methods section, we formally describe the three approaches and the simulations we performed to assess the performance of these approaches. We also describe a dataset from the EPIC study on which we illustrate the methods. In the Results, we present a theoretical investigation of the properties of JAV, showing that although JAV gives consistent estimation for linear regression under MCAR, it will not, in general, under MAR. Results from the simulations and from applying the methods to the EPIC dataset are also described there. These results are followed by a discussion and conclusions.

## Methods

### Three imputation methods

We begin by describing passive imputation, PMM and JAV for the setting of linear regression of *Y *on *X *and *X*^2^. We then describe the modifications necessary for regression of *Y *on *X, Z *and *XZ*.

Let *X_i _*and *Y_i _*denote the values of *X *and *Y*, respectively, for subject *i *(*i *= 1,...,*n*). Assume that (*X*_1_, *Y*_1_),...,(*X_n_, Y_n_*) are independently identically distributed. Let *R_i _*= 1 if *X_i _*is observed (i.e. if subject *i *is a complete case), with *R_i _*= 0 otherwise. Let *n*_1 _denote the number of complete cases, and *q *denote the number of regression parameters in the imputation model. Let $\bar{X}={\left(X_{1},\ldots ,X_{n}\right)}^{T}$, ***W**_i_*=*R_i _*(1, *Y_i_*)*^T ^*(so ***W ***= (0, 0)*^T ^*whenever *X *is missing), $\bar{W}={\left(W_{1},\ldots ,W_{n}\right)}^{T}$, and $\psi ={\left({\bar{W}}^{T}\bar{W}\right)}^{-1}$.

### Passive imputation

In the approach we call 'linear imputation model with passive imputation of *X*^2^' (or just 'passive imputation') the linear regression model $X\sim N\left(\gamma_{0}+\gamma_{1}Y,\sigma^{2}\right)$ is fitted to the complete cases. So, *q *= 2. Let $\hat{\gamma }=\left({\hat{\gamma }}_{0},{\hat{\gamma }}_{1}\right)=\psi {\overset{¯}{W}}^{T}\bar{X}$ denote the resulting maximum likelihood estimate (MLE) of $\gamma =\left(\gamma_{0},\gamma_{1}\right)$, and let σ^2= ∑i=1nRiXi-γ^TWi2/n1-q denote the unbiased estimator of *σ*^2^. If  and *σ*^2 ^are treated as a priori independent with joint density proportional to *σ*^-2^, then the posterior distribution of n1-qσ^2/σ2 is $\chi_{n_{1}-q}^{2}$ and that of  given *σ*^2 ^is $N\left(\hat{\gamma },\;\psi \sigma^{2}\right)$[6]. So, to create a single imputed dataset, $\sigma^{*2}$ is drawn from n1-qσ^2/χn1-q2 and $\gamma^{*}$ from $N\left(\hat{\gamma },\;\psi \sigma^{*2}\right)$. Then missing *X *values are imputed as $X_{i}=\gamma^{*T}W_{i}+\sigma^{*}B_{i}$, where the *B_i_*'s are independently distributed *N*(0, 1).

### PMM

The approach we call 'linear imputation model with predictive mean matching' (or just 'PMM') is the same as passive imputation up to the generation of $\sigma^{*2}$ and $\gamma^{*}$. Thereafter, instead of generating $\gamma^{*T}W_{i}+\sigma^{*}B_{i}$, a fitted value X^i*=γ*TWi is calculated for each subject. For each subject *i *with missing *X*, the *K *subjects with observed *X_j _*and the closest X^j* values to his or her X^i* value are identified. One of these *K *subjects is chosen at random and his or her *X_j _*value becomes the imputed value of *X_i_*. The square of the imputed value of *X_i _*becomes the imputed value of $X_{i}^{2}$. The value of *K *is chosen to balance bias in parameter and variance estimation. If *K *is very large, matching is very loose, leading to bias in parameter estimates of the analysis model. If *K *is very small, uncertainty in the imputed data will not be fully represented, leading to underestimation of standard errors when Rubin's Rules are applied. For our simulations we used *K *= 5. Notice that if, as in this case, the imputation model is a simple linear regression of *X *on *Y*, finding the subjects with the nearest X^j* values to X^i* is equivalent to finding the subjects with the nearest *Y_j _*values to *Y_i_*. If the imputation model contains more than one predictor, PMM may be quite different from matching on the subjects with the nearest *Y_j_*.

### JAV

In the JAV approach, (*Y, X, X*^2^) is assumed to be jointly normally distributed:

(1) $$\left[\begin{array}{c} Y\\ X\\ X^{2} \end{array}\right]∼N\left(\left[\begin{array}{c} \mu_{1}\\ \mu_{2}\\ \mu_{3} \end{array}\right],\;\left[\begin{array}{ccc} \sigma_{11}\; & \sigma_{12}\; & \sigma_{13}\\ \sigma_{12}\; & \sigma_{22}\; & \sigma_{23}\\ \sigma_{13}\; & \sigma_{23}\; & \sigma_{33} \end{array}\right]\right).$$

Expression (1) can equivalently be written as

(2) $$Y\sim N\left(\mu_{1},\sigma_{11}\right)$$

(3) $$\left[\begin{array}{l} X\\ X^{2} \end{array}\right]|Y∼N\left(\left[\begin{array}{l} \delta_{20}+\delta_{21}Y\\ \delta_{30}+\delta_{31}Y \end{array}\right],\left[\begin{array}{l} \tau_{22}\;\tau_{23}\\ \tau_{23}\;\tau_{33} \end{array}\right]\right).$$

where (for *k *= 2, 3) $\delta_{k0}=\mu_{k}-\mu_{1}\sigma_{12}/\sigma_{11}$, $\delta_{k1}=\sigma_{12}/\sigma_{11}$, $\tau_{kk}=\sigma_{kk}-\sigma_{1k}^{2}/\sigma_{11}$ and $\tau_{12}=\sigma_{23}-\sigma_{12}\sigma_{13}/\sigma_{11}$. Having fitted model (1) to the observed data, a perturbation is added to the maximum likelihood estimates, in a similar way to that described above for the passive imputation method. Missing values of *X *and *X*^2 ^are then generated from distribution (3) using the perturbed values of the parameters. As *Y *is fully observed, an alternative to fitting model (1) is just to fit model (3) directly.

The methods described above need only minor adaption for the setting of linear regression of *Y *on *X, Z *and *XZ*. In passive imputation and PMM, the imputation model for *X *should include *Z*. Obvious choices are $X\sim N\left(\gamma_{0}+\gamma_{1}Y+\gamma_{2}Z,\sigma^{2}\right)$ (so $W_{i}=R_{i}{\left(1,Y_{i},Z_{i}\right)}^{T}$ and *q *= 3) or $X\sim N\left(\gamma_{0}+\gamma_{1}Y+\gamma_{2}Z+\gamma_{3}YZ,\sigma^{2}\right)$ (so $W_{i}=R_{i}{\left(1,Y_{i},Z_{i},Y_{i}Z_{i}\right)}^{T}$ and *q *= 4). The imputed value of *X *multiplied by the imputed individual's value of *Z *becomes the imputed value of *XZ*. In JAV, (*Y, X, Z, XZ*), rather than (*Y, X, X*^2^), is assumed to be multivariate normally distributed.

### Simulation studies

#### Linear regression with quadratic term

**Figure 1:**
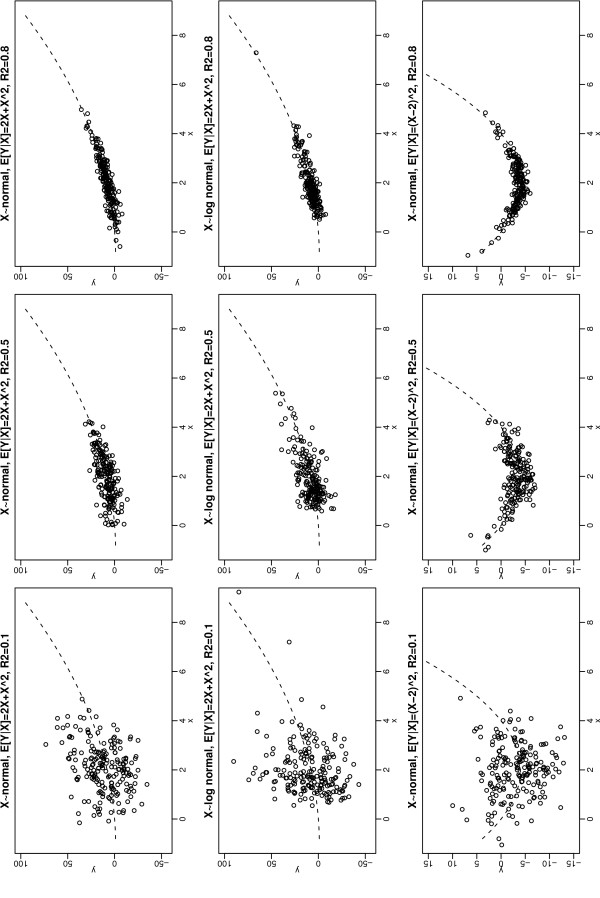
**Typical datasets for normally or log-normally distributed *X *(each with mean 2 and variance 1), normally distributed *Y *with mean 2*X *+ *X*^2 ^or (*X *- 2)^2^, and *R*^2 ^= 0.1, 0.5 or 0.8**. Dotted line shows expected value of Y given *X*.

In all our linear regression simulation studies, a sample size of 200 was assumed and 1000 simulated datasets were created. For each simulated dataset, we generated 200 *X *values from one of four distributions with mean 2 and variance 1: normal, log normal, (shifted and scaled) beta, and uniform. For the log normal distribution, *logX *was generated from $N\left(\log\left(\sqrt{3.2}\right),\log\left(5/4\right)\right)$; *X *then has a coefficient of skewness of 1.63. For the (shifted and scaled) beta distribution, we generated *Z *~ beta(1, 10) and *X*= 12.05(*Z*-1/11)+2; *X *then has a skewness of 1.51. The outcome *Y *was generated from *N*(2*X*+*X*^2^,*ϕ*), where *ϕ *was chosen to make the coefficient of determination *R*^2 ^equal to 0.1, 0.5 or 0.8. Although *R*^2 ^values greater than 0.5 are uncommon in medical studies, we wanted also to investigate the performance of methods in extreme situations. The top two rows of Figure 1 show, for normally and log-normally distributed *X*, a typical set of data generated in this way.

Missingness was then imposed on these data. Let expit(*x*) = {1+exp(-*x*)}^-1^. *Y *was fully observed; two missing data mechanisms were assumed for *X*. For MCAR, each *X *was observed with probability 0.7, regardless of the values of *X *and *Y*. For MAR, the probability *X *was observed was expit(*α*_0 _+ *α*_1_*Y*), where α_1_=-1/SD(*Y*) and *α*_0 _was chosen to make the marginal probability of observing *X *equal to 0.7.

For the three methods, passive imputation ('Passive'), PMM and JAV, we used *M *= 5 imputations. We also carried out the complete-case analysis ('CCase') and the complete-data analysis ('CData'), i.e. before data deletion.

Finally, we instead generated *Y *from *N *((*X*-2)^2^,*ϕ*), with *ϕ *chosen to make *R*^2 ^= 0.1, 0.5 or 0.8. As the mean of *X *is 2, the quadratic relation between *Y *and *X *is now more obvious in such data (see Figure 1).

### Linear regression with interaction

We focussed on normally and log-normally distributed covariates. Four bivariate distributions were assumed for the two covariates *X *and *Z*. In the first, *X *and *Z *were both independently distributed *N*(2, 1). In the second, they were generated from a bivariate normal distribution so that they both had marginal distribution *N*(2, 1) but Cor(*X, Y*) = 0.5. In the third, *logX *and *logZ *were independently distributed $N\left(\log\left(\sqrt{3.2}\right),\log\left(5/4\right)\right)$, so that *X *and *Z *were independently log-normal each with mean 2 and variance 1. In the fourth, *logX *and *logZ *were generated from a bivariate normal distribution so that they both had marginal distribution $N\left(\log\left(\sqrt{3.2}\right),\log\left(5/4\right)\right)$ but Cor((log *X*, log *Z*) = 0.5. Outcome *Y *was generated from *N *(*X+Z+XZ, ϕ*), where *ϕ *was chosen so that *R*^2 ^= 0.1 or 0.5.

*Y *and *Z *were fully observed; the same two missing data mechanisms were assumed for *X *as in 'Linear regression with Quadratic Term'. Two variations of passive imputation were used: in the first ('Passive1'), the imputation model contained just *Y *and *Z*; in the second ('Passive2'), the imputation also contained the interaction *YZ*. For PMM the imputation model also included *YZ*. Von Hippel [3] considered only Passive1.

### Logistic regression with quadratic term

A sample size of 2000 was assumed and 1000 simulated datasets were created. This larger sample size was used because binary outcomes provide less information for estimating parameter values than do continuous outcomes. We used the same normal and log normal distributions for *X *as in 'Linear regression with Quadratic Term'. Binary outcomes *Y *were generated from the model *P*(*Y *= 1|*X*) = expit(*β*_0_+2*β*_2_*X*+*β*_2_*X*^2^). The value of *β*_2 _was chosen to make the log odds ratio of *Y *for *X *= 3 versus *X *= 1 equal to either 1 (*β*_2 _= 1/12) or 2 (*β*_2 _= 1/6). When *X *is normally distributed, this is the log odds ratio for the mean of *X *plus one standard deviation relative to the mean of *X *minus one standard deviation. The value of *β*_0 _was chosen so that the marginal probability of *Y *= 1 was either *p *= 0.1 or *p *= 0.5.

*Y *was fully observed; *X *was MCAR or MAR, with probability expit(*α*_0 _+ *α*_1_*Y*) of being observed. For MCAR *α*_1 _= 0; for MAR *α*_1 _= - 2. In both cases *α*_0 _was chosen to give a marginal probability of observing *Y *of 0.7. For passive imputation and PMM the imputation model was the linear regression of X on Y.

### Analysis of vitamin C data from EPIC Study

EPIC-Norfolk is a cohort of 25,639 men and women recruited during 1993-97 from the population of individuals aged 45-75 in Norfolk, UK [7]. Shortly after recruitment, study participants were invited to attend a health check at which a 7-day diet diary was provided for completion over the next week. Blood samples were provided and have been stored. A measure of average daily intake of vitamin C has been derived from the 7-day diet diary and plasma vitamin C (*μ*mol/l) was measured within a few days of the blood sample being provided. The dietary assessment methods have been described in detail elsewhere [8].

There is evidence of a non-linear association between vitamin C intake and plasma vitamin C [9]. Here, we look at this association in the EPIC-Norfolk data: in particular, whether this relation is linear or has a quadratic element. Plasma vitamin C is also affected by sex, age, smoking status, and body size [9-12], so these possible confounders are adjusted for in our analysis. The analysis presented in this article illustrates the methods described here and is not intended as a definitive analysis of the EPIC data.

Of the 25639 subjects, 10224 had incomplete data: 3165 had missing plasma vitamin C; 8100 missing dietary vitamin C; 32 missing weight; 220 missing smoking status (age and sex were fully observed). If the data are not MCAR, estimators from a complete-case analysis may be biased. When logistic regression was used on the set of subjects with observed plasma vitamin C, higher values of log plasma vitamin C were associated with a lower probability of being a complete case (*p *= 0.03). This suggests the data are not MCAR. Furthermore, the complete-case analysis ignores information on individuals with observed outcome plasma vitamin C but one or more missing covariates. For these two reasons, we applied MI to these data.

Three forms of MI were used. In the first and second forms, the full-conditional specification (FCS: also known as 'chained-equations') approach was used [13]. This involves cycling through the variables, imputing missing values in each variable in turn using a model for the distribution of that variable given all the other variables except vitamin C-squared. In the first form of MI, a linear regression model was used to impute each variable except dietary vitamin C-squared and smoking status. Multinomial logistic regression was used for smoking status and dietary vitamin C-squared was passively imputed from dietary vitamin C. If all variables except dietary vitamin C had been observed, this approach would be the same as what we called 'linear imputation model with passive imputation of *X*^2^' in the simulations. The second form of MI was identical to the first except that PMM was used to impute dietary vitamin C. The third form of MI was JAV, i.e. imputation under a multivariate normal distribution. For JAV, smoking status was represented by two binary indicator variables, one of which was equal to 1 for former smokers, the other of which equalled 1 for never smokers. Imputed values for these binary variables were not rounded. This method of handling categorical variables was advocated by Ake [14] and, in view of the small proportion of missing values in the smoking variable, should be adequate. For all three MI methods, the 3165 subjects with missing plasma vitamin C were used for imputation but were deleted from the dataset before fitting the analysis model. When, as in this case, the same set of variables are used in the imputation as in the analysis, subjects with missing responses can provide information about the joint distribution of the covariates and hence about missing covariate values in subjects with observed responses, but do not otherwise carry information about the parameters of the analysis model [15]. Deletion of such subjects after imputation reduces the Monte Carlo error caused by having a finite number of imputed datasets. Standard errors were estimated for each imputed dataset using the robust variance estimator [16], as there was evidence of heteroskedacity (see Results).

We also applied a variant of JAV in which FCS was used. This variant was identical to the first form of MI except that the dietary vitamin C-squared variable was imputed using a linear regression model involving all the other variables (including dietary vitamin C) as covariates. This is equivalent to imputing dietary vitamin C and dietary vitamin C-squared from a bivariate normal distribution conditional on all the other variables.

In all our analyses smoking status was categorised as current smoker (baseline), former smoker or never smoker. The first and second forms of MI and the variant JAV method were implemented using ice in STATA. The original JAV method was implemented using mi impute mvn in STATA.

## Results

### Properties of JAV under MCAR and MAR

In this section, we summarise the argument of Von Hippel (2009) for why the JAV approach will give consistent estimation of the parameters of the analysis model when the data are MAR, and then explain why JAV actually requires the stronger condition of MCAR for consistency.

Assume that the analysis model is the regression of *Y *on *X *and *X*^2 ^(the argument is analogous for the interaction model). Model (1), or equivalently (2)-(3), is misspecified, since (*Y, X, X*^2^) is not joint normally distributed. The values of *μ*_1_, *σ*_11_, etc. that minimise the Kullback-Leibler distance between the true distribution of (*Y, X, X*^2^) and the multivariate normal distribution are called their 'least false' values [17]. The least false values of *μ*_1_, *μ*_2 _and *μ*_3 _are just the population means of *Y, X *and *X*^2^; those of *σ*_11_,..., *σ*_33 _are the population variances and covariances. If the missing *X *and *X*^2 ^values are imputed from distribution (3) with **Δ **= (*δ*_20_,*δ*_21_, *δ*_30_, *δ*_31_, *τ*_22_, *τ*_23_, *τ*_33_,) equal to its least false value, then the mean and variance of (*Y, X, X*^2^) in the imputed dataset will consistently estimate the mean and variance in the population. The true parameter values of the analysis model are functions of this population mean and variance: they are ${\left\{E\left(U_{1}U_{1}^{T}\right)\right\}}^{-1}E\left(U_{1}Y_{1}\right)$, where $U_{1}={\left(1,X_{1},X_{1}^{2}\right)}^{T}$. Therefore, if missing *X *and *X*^2 ^values are imputed using the least false values, the parameters of the analysis model will be consistently estimated.

Von Hippel argues that, when *X *is MAR, the least false values of Δ can be consistently estimated from the observed data. Von Hippel proposes that therefore the missing data can be imputed using the assumption that (*Y, X, X*^2^) is jointly normally distributed (model (1)). He does this using PROC MI in SAS.

When the data are MCAR, the above argument is valid. However, when the data are MAR, the observed-data MLEs of **Δ **are not necessarily consistent for the least false value, because model (1) is misspecified. Consequently, the analysis model parameters will not, in general, be consistently estimated unless *X *is MCAR. In more detail, the argument is as follows.

It can be seen from expressions (2) and (3) that the MLEs of *μ*_1 _and *σ*_11 _are functions only of the *Y *values of everyone in the sample and do not depend on *X*, while the MLE of **Δ **is a function of the *Y, X *and *X*^2 ^values of subjects for whom *X *is observed. As *Y *is fully-observed, the MLEs of *μ*_1 _and *σ*_11 _consistently estimate their least false values. If *X *is MCAR, the set of individuals for whom *X *is observed is a simple random subsample from the sample, and hence also a simple random sample from the population. So, **Δ **is also consistently estimated by its MLE. The rest of Von Hippel's argument now applies. On the other hand, if *X *is MAR, the individuals with observed *X *are not a simple random subsample of the sample, and hence represent a random sample from another population different from that from which the actual sample was drawn. In this other population, the least false value of Δ will generally be different [17,18]. The maximum likelihood estimator of Δ will consistently estimate the least false value in this other population. It follows that, in general, JAV will give inconsistent estimation of the parameters in the analysis model.

The argument that JAV will give consistent estimation when *X *is MCAR can be straightforwardly extended to allow for a vector of additional variables, ***S ***say. In this case, the normal distribution for (*Y, X, X*^2^) in equation (1) is extended to a normal distribution for (*Y, X, X*^2^, ***S***). ***S ***may include both variables that are additional covariates in the analysis model and variables that are not in the analysis model (i.e. auxiliary variables). If (*X, **S***) are MCAR, JAV will give consistent estimation of the parameters of the analysis model.

So far, we have been concerned with parameter estimation. Now consider variance estimation. Von Hippel uses Rubin's Rules to estimate variances and hence confidence intervals. However, there is no particular reason to assume that Rubin's Rules will give a consistent variance estimator, because derivations of Rubin's Rules assume a correctly specified parametric imputation model [19-21] and the multivariate normal model is misspecified. In our simulations, we investigate both the bias in parameter estimators and the coverage of confidence intervals calculated using Rubin's Rules.

### Simulation studies

#### Linear regression with quadratic term

We focus on the quadratic term, whose true value is 1. The first block of five rows of Table 1 shows the bias and 95% coverage for the five methods when *X *is normally distributed and MCAR. Also shown is the relative precision, i.e. the ratio of the empirical variance of CData to those of the other estimators. When both estimators contributing to this ratio are unbiased, it is the relative efficiency. The maximum (over the five methods) Monte Carlo standard errors (MCSE) associated with the estimated biases are reported in the table legend. The estimated biases of CData (-3%, -1%, 0%; MCSEs 3%, 1% and 1%) and CCase (-2%, -1%, 0%; MCSEs 4%, 1%, 1%) are consistent with these estimators being unbiased, as is expected. CData and CCase both have estimated coverage 95%, again as expected. Due to Monte Carlo variation, the variance of CData is slightly less than the expected 0.7 times that of CCase. JAV is unbiased, as expected; its coverage is approximately correct. JAV is, however, slightly less efficient than CCase. This difference in efficiency narrowed to 1% when the number of imputations was increased to *M *= 30 (data not shown). Note that if other non-fully observed variables, *V *say, were included as covariates in the analysis model along with *X *and *X*^2^, JAV might well be more efficient than CCase, because CCase would not use data on individuals with observed *X *but missing *V*, whereas JAV would. Passive underestimates the quadratic effect by 20-30%; coverage is too high when *R *= 0.1 and too low when *R *= 0.8. PMM is approximately unbiased, but may have slight under-coverage. The underestimation by Passive of the quadratic effect accords with the finding of Von Hippel (2009), who applied Passive and JAV to a real dataset. He found that the estimate of the quadratic effect from Passive was closer to zero than that from JAV.

**Table 1:** Linear regression with *Y *~ *N *(2*X*+*X*^2^, *ϕ*)

	***R***^**2 **^**= 0.1**			*R*^2 ^= 0.5			*R*^2 ^= 0.8		
	bias	cover	**r.prec**.	bias	cover	**r.prec**.	bias	cover	**r.prec**.
	MCAR, *X *~ normal								
CData	-3	95	100	-1	95	100	0	95	100
CCase	-2	95	64	-1	95	64	0	95	64
Passive	-32	99	124	-21	95	104	-20	87	86
PMM	-3	92	59	0	93	65	2	92	64
JAV	-4	94	61	-1	95	61	0	95	62
	MAR, *X *~ normal								
CData	-6	95	100	-1	96	100	-2	95	100
CCase	-23	95	72	-13	95	59	-8	94	48
Passive	-45	99	144	-27	95	120	-42	50	122
PMM	-36	89	50	-13	93	49	8	91	36
JAV	-12	94	52	-1	95	42	0	93	38
	MAR, *X *~ log normal								
CData	-6	96	100	0	95	100	-1	95	100
CCase	-21	94	42	-19	94	24	-7	94	20
Passive	-72	98	70	24	93	21	-3	88	31
PMM	-46	88	29	-19	90	15	47	86	6
JAV	-7	92	28	7	91	12	18	91	10

Table 1 Percentage bias, coverage and relative precision for quadratic term in linear regression when *Y *~ *N *(2*X*+*X*^2^, *ϕ*). The true value of the quadratic term is 1. For MCAR, *X *~ normal, the maximum MCSEs among the five methods are 4, 1 and 1% for *R*^2 ^= 0.1, 0.5 and 0.8, respectively. For MAR, *X *~ normal, they are 5, 2 and 1%. For MAR, *X *~ log normal, they are 7, 4 and 3%

The above observations remain broadly true when *X *is log-normal, beta or uniform (data not shown).

The second block of five rows of Table 1 shows bias, coverage and relative precision when *X *is normally distributed and MAR. Unlike in the MCAR case, CCase, PMM and JAV are now biased. Of the (non-complete data) methods, JAV has the smallest bias, and in no case is it greater than 12% (MCSE 4%); its coverage is approximately correct. We do not discuss relative precisions: they are not very meaningful in the presence of bias. The bias in PMM probably arises because the largest missing value (or values) of *X *tends to be larger than the largest observed value of *X*, because the probability that *X *is observed decreases as *Y *increases and individuals with larger *Y *values tend to have larger *X *values. This means that the imputed values of *X *tend to be lower than their corresponding missing true values.

The third block of five rows of Table 1 shows the results when *X *is log-normally distributed and MAR. When *R*^2 ^= 0.1, CCase is biased and JAV approximately unbiased. However, when *R*^2 ^= 0.8, JAV is considerably more biased than CCase. There is some evidence of slight undercoverage of JAV. When *X *is beta distributed, JAV is approximately unbiased when *R*^2 ^= 0.1, but biased (bias = 20%) when *R*^2 ^= 0.8. JAV is approximately unbiased with coverage 93% when *X *is uniformly distributed (data not shown). PMM had large bias for all three values of *R*^2^.

Increasing the sample size to *N *= 5000 reduces the bias (to < 4%) of PMM when *X *is normally distributed and MAR (data not shown). This is probably because a larger sample size enables closer matches to be found for individuals with missing *X*. However, there was no consistent improvement when *X *was log-normally distributed. Although the bias reduced from -46% to -12% when *R*^2 ^= 0.1, it stayed the same when *R*^2 ^= 0.8 and increased from -19% to 38% when *R*^2 ^= 0.5. The biases of Passive and JAV are not improved. Coverage of PMM, Passive and JAV worsen, especially when *R*^2 ^= 0.8 or *X *is log-normally distributed: when *X *is normally distributed and *R*^2 ^= 0.8, coverages were 0%, 67% and 88%, respectively. We also investigated whether the bias of PMM that was still evident for a sample size of *N *= 5000 when *X *was log normally distributed was reduced by increasing *N *to 50000. We found that, although this did happen, the bias was still 15% when *R*^2 ^= 0.5 and 26% when *R*^2 ^= 0.8. This is probably because the difference between the largest missing value (or values) of *X *and the largest observed value of *X *could continue to be quite large, even with a very large sample size, when *X *is log normally distributed and the missingness mechanism means that very large values of *X *are very likely to be missing.

Table 2 shows the results when *Y *~ *N *((*X*-2)^2^, *ϕ*). JAV is approximately unbiased when *X *is MCAR. Estimated coverage is at least 92% when *X *is normally or uniformly distributed. However, when *R*^2 ^= 0.8 and *X *is MCAR and log-normally or beta distributed, coverage is only 83% (data not shown). The coverage deteriorates as the sample size increases: 62%, 71% and 62% when *N *= 5000 and *X *is log-normally distributed and *R*^2 ^= 0.1, 0.5 or 0.8, respectively. When *X *is MAR and normally distributed, the bias of JAV is 18% (coverage 68%) when *R*^2 ^= 0.5 and 22% (coverage 19%) when *R*^2 ^= 0.8. The bias is 41% or 71% when *X *is MAR and log-normally distributed with *R*^2 ^= 0.5 or 0.8, respectively. These biases do not improve as sample size increased. The performances of JAV and PMM are similar when *X *is MCAR; JAV can be better or worse than PMM when *X *is MAR. When the sample size is increased and *X *is normally distributed, the bias of PMM diminishes (the maximum bias of PMM when *N *= 5000 is 6%), but that of JAV does not; when *X *is log-normally distributed, neither bias diminishes for all values of *R*^2^.

**Table 2:** Linear regression with *Y *~ *N *((*X*-2)^2^, *ϕ*)

	***R***^**2 **^**= 0.1**			*R*^2 ^= 0.5			*R*^2 ^= 0.8		
	bias	cover	**r.prec**.	bias	cover	**r.prec**.	bias	cover	**r.prec**.
	MCAR, *X *~ normal								
CData	-1	95	100	0	95	100	0	95	100
CCase	0	95	64	0	95	64	0	95	64
Passive	-31	86	110	-31	48	55	-30	32	21
PMM	-2	93	62	1	93	61	2	90	47
JAV	-1	94	64	0	93	63	0	92	52
	MAR, *X *~ normal								
CData	0	94	100	0	95	100	0	94	100
CCase	-14	92	54	-9	88	37	-4	91	30
Passive	-41	80	108	-38	45	52	-32	48	18
PMM	-10	88	42	4	88	26	16	51	10
JAV	0	93	41	18	68	21	22	19	10
	MAR, *X *~ log normal								
CData	2	94	100	0	95	100	0	95	100
CCase	-12	94	44	-8	94	27	-4	94	20
Passive	-41	96	81	-25	87	18	-9	90	4
PMM	-10	88	29	8	91	12	35	70	3
JAV	7	92	27	41	70	6	71	20	2

Table 2 Percentage bias, coverage and relative precision for quadratic term in linear regression when *Y *~ *N *((*X*-2)^2^, *ϕ*). For MCAR, *X *~ normal, the maximum MCSEs among the five methods are 1, 0 and 0% for *R*^2 ^= 0.1, 0.5 and 0.8, respectively. For MAR, *X *~ normal, they are 1, 1 and 1%. For MAR, *X *~ log normal, they are 2, 2 and 2%

### Linear regression with interaction

We focus on the interaction term, whose true value is 1. Table 3 shows the results when *X *and *Z *are independent. It can be seen that Passive1 is heavily biased even when *X *is MCAR. Including the *YZ *term in the imputation model (Passive2) usually, but not always reduces the bias, but it remains substantial. PMM offers little or no improvement over Passive 2. JAV performs much better, being approximately unbiased. Coverage of JAV is good, although there is evidence of slight undercoverage: its coverage is consistently less than that of CCase when *X *is MAR. The underestimation by Passive of the interaction effect accords with the finding of Von Hippel (2009) in a real dataset that the estimate from Passive was closer to zero than that from JAV.

**Table 3:** Linear regression with interaction

	***R***^**2 **^**= 0.1**			*R*^2 ^= 0.5			*R*^2 ^= 0.8		
	bias	cover	**r.prec**.	bias	cover	**r.prec**.	bias	cover	**r.prec**.
	MCAR, *X, Z *~ normal								
CData	3	93	100	1	93	100	0	93	100
CCase	-3	95	71	-1	95	71	0	95	71
Passive1	-31	97	136	-19	94	116	-18	88	106
Passive2	-11	95	86	-17	94	115	-17	89	103
PMM	-12	96	86	-15	96	106	-13	91	93
JAV	-2	93	66	-1	94	65	0	94	65
	MAR, *X, Z *~ normal								
CData	-1	94	100	-2	95	100	0	95	100
CCase	-15	96	82	-12	94	69	-5	95	62
Passive1	-36	99	147	-24	94	112	-25	79	110
Passive2	-14	96	75	-26	94	111	-25	82	89
PMM	-19	97	84	-23	94	94	-17	90	85
JAV	-3	94	60	-4	92	54	1	94	53
	MAR, *X, Z *~ log normal								
CData	-1	96	100	2	95	100	1	96	100
CCase	-17	94	57	-9	95	38	-4	96	30
Passive1	-43	98	129	-20	96	76	-34	68	65
Passive2	-40	96	71	-42	89	58	-45	73	27
PMM	-40	96	79	-38	92	66	-27	85	30
JAV	-3	93	41	8	92	26	14	92	20

Table 3 Percentage bias, coverage and relative precision for interaction term in linear regression. The true value of the interaction term is 1. For MCAR, *X, Z *~ normal, the maximum MCSEs are 4, 1 and 1% for *R*^2 ^= 0.1, 0.5 and 0.8, respectively. For MAR, *X, Z *~ normal, they are 4, 2 and 1%. For MAR, *X, Z *~ log normal, they are 6, 3 and 1%

When *X *and *Z *are correlated (results not shown), the biases of passive imputation and PMM change, but remain larger than those of JAV, whose biases (and coverages) change little. PMM is still less biased than passive imputation when *X *is MCAR, but not necessarily when *X *is MAR. For example, when *X *and *Z *are (marginally) normally distributed and *R*^2 ^= 0.1, the biases of Passive1, Passive2 and PMM are 24%, -16% and 24%, respectively; when *X *and *Z *are log normally distributed and *R*^2 ^= 0.1, they are -29%, -37% and -64%.

### Logistic regression with quadratic term

We focus on the quadratic term, whose true value is *β*_2 _= 1/12 or 1/6. Table 4 shows the results. Consider first the results when *X *is MCAR. Passive has large bias. PMM is approximately unbiased, but slightly less efficient than CCase. In fact, because *Y *is binary, and so each missing *X *value is replaced by an observed *X *from a randomly chosen individual with the same value of *Y*, PMM is equivalent to CCase but with each complete case receiving a random weight. As these weights are (over repeated samples) uncorrelated with *Y *and *X*, they do not cause bias, but do introduce stochastic variation that is not present in CCase. When *p *= 0.5, *β*_2 _= 1/2 and *X *is normally distributed, JAV performs well. This is in conformity with the hypothesis of Von Hippel: *P*(*Y *= 1|*X*) is fairly close to 0.5 over most of the distribution of *X*, and so the logistic function is an approximately linear function of the linear predictor over most of the distribution of *X*. When *p *= 0.1, *β*_2 _= 1/6 or *X *is log normally distributed, however, JAV performs much worse and in one case changes direction: now *P*(*Y *= 1|*X*) is closer to zero or one for more individuals in the population.

**Table 4:** Logistic regression with quadratic term

**(*p, β***_**2**_)	(0.5, 1/12)			(0.5, 1/6)			(0.1, 1/12)		
	bias	cover	**r.prec**.	bias	cover	**r.prec**.	bias	cover	**r.prec**.
	MCAR, *X *~ normal								
CData	1	95	100	-1	95	100	-6	94	100
CCase	1	96	70	-1	95	73	-8	95	67
Passive	-30	97	137	-30	92	136	-34	99	119
PMM	0	94	67	-1	94	70	-10	93	63
JAV	-7	96	76	-23	92	102	27	91	72
	MCAR, *X *~ log normal								
CData	6	95	100	4	94	100	4	94	100
CCase	7	94	69	4	95	73	4	96	71
Passive	-36	96	222	-45	90	308	-40	93	127
PMM	8	93	67	6	92	68	5	95	68
JAV	-66	71	178	-118	3	398	55	85	56
	MAR, *X *~ normal								
CData	0	96	100	1	95	100	-8	96	100
CCase	-1	97	67	0	95	63	-28	96	28
Passive	33	97	125	-30	92	115	-71	99	171
PMM	-2	94	65	-1	92	59	-33	85	27
JAV	37	89	59	56	62	79	51	82	26
	MAR, *X *~ log normal								
CData	5	93	100	5	96	100	5	94	100
CCase	7	93	70	7	95	69	7	95	38
Passive	-8	98	100	2	99	106	-202	16	81
PMM	8	91	67	7	93	64	5	84	34
JAV	22	92	81	-30	80	105	333	25	7

Table 4 Percentage bias, coverage and relative precision for quadratic term in logistic regression. For MCAR, *X *~ normal, the maximum MCSEs are 2, 1 and 3% for (*p, β*_2_)=(0.5,1/12), (0.5, 1/6) and (0.1, 1/12), respectively. For MCAR, *X *~ log normal, they are 2, 2 and 2%. For MAR, *X *~ normal, they are 2, 1 and 4%. For MAR, *X *~ log normal, they are 2, 2 and 6%

Now consider the results when *X *is MAR. When *p *= 0.5, the CCase is approximately unbiased with correct coverage. This is because the complete cases can be regarded as a sample from a case-control study: the probabilities that individuals are sampled depends on their outcomes (*Y*) but not their exposures (*X*). As is well known, valid inference can be obtained from case-control study data using ordinary logistic regression and treating *Y *as the outcome [22]. PMM is approximately unbiased with correct coverage for the same reason. Note that when *p *= 0.1, *β*_2 _= 1/12 and *X *is normally distributed, even CCase and PMM are subject to finite sample bias. This is because although the sample size is 2000, and so the expected number of cases is 200, the expected number with observed *X *is only 47 under this MAR mechanism. This would seem to be too few to unbiasedly estimate the quadratic effect. The bias of JAV can be very large: up to 56% when *X *is normally distributed and 333% when *X *is log normally distributed. Unsurprisingly, its coverage can also be very poor.

### Analysis of vitamin C data from EPIC study

**Figure 2:**
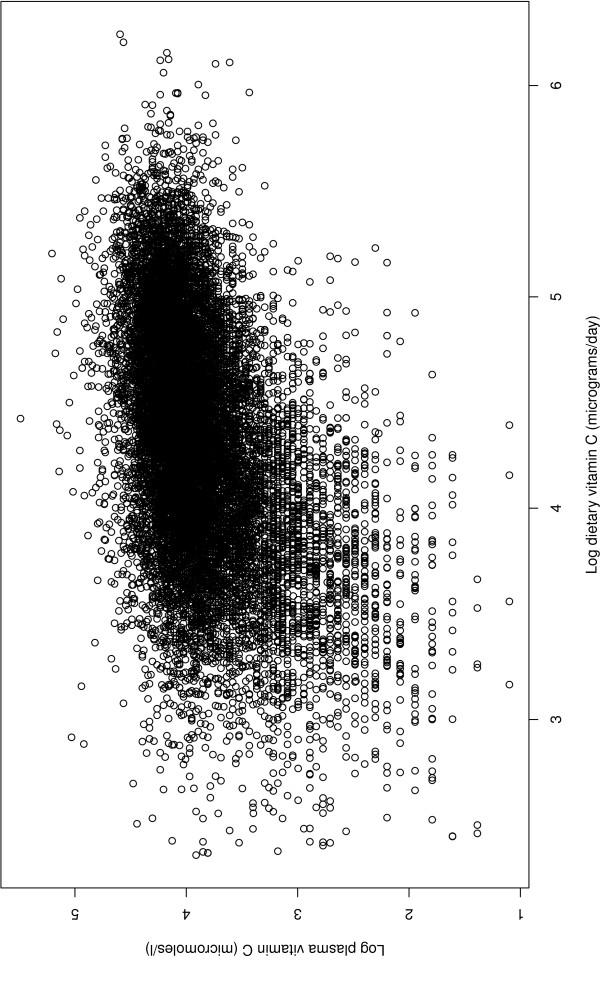
**Log plasma vitamin C and log dietary vitamin C in 15415 individuals for whom both variables are observed**.

Figure 2 is a plot of log plasma vitamin C (*μ*mol/l) against log dietary vitamin C (mg/day). There is a suggestion of a quadratic effect: the gradient appears to diminish as the dietary vitamin C value increases. Table 5 shows the estimates from a linear regression in the complete cases of log plasma vitamin C on log dietary vitamin C and the confounders. Smoking status is categorised as current smoker (baseline), former smoker or never smoker. As Figure 2 shows, there is evidence of heteroskedasticity. The quadratic effect is highly significant and negative, according with the apparent diminishing gradient.

**Table 5:** Analysis of vitamin-C data

	Complete		FCS		FCS		JAV	
	Cases		with Passive		with PMM			
	Est	SE	Est	SE	Est	SE	Est	SE
intercept	0.990	0.201	0.570	0.177	0.903	0.181	1.030	0.163
log diet C	1.141	0.090	1.322	0.079	1.163	0.081	1.106	0.075
log diet C sqrd	-0.090	0.010	-0.113	0.009	-0.094	0.009	-0.088	0.008
sex	0.169	0.008	0.173	0.007	0.172	0.007	0.172	0.007
weight (per 10 Kg)	-0.042	0.003	-0.041	0.003	-0.040	0.003	-0.041	0.003
age (per 10 yrs)	-0.052	0.004	-0.043	0.003	-0.043	0.003	-0.043	0.003
former smoker	0.212	0.015	0.213	0.012	0.213	0.012	0.212	0.012
never smoker	0.216	0.014	0.218	0.012	0.218	0.012	0.219	0.012

Table 5 Point estimates and SEs from complete-case analysis and three MI methods (full conditional specification with and without predictive mean matching, and JAV) for the regression of log plasma vitamin C on log dietary vitamin C ('log diet C'), its square ('log diet C sqrd') and a set of confounders

Table 5 shows the estimates for three of the four MI methods. The results from the variant JAV method are almost identical to those from JAV, and so are not reported here. It can be seen that the point estimates from JAV are very similar to those from the complete-case analysis. The estimates from the FCS with passive imputation method are quite different from those of JAV: estimated quadratic and linear effects of log dietary vitamin C are stronger. This difference is much reduced when PMM is used instead of passive imputation, suggesting that the linear model used to impute missing dietary vitamin C values in the passive method may have induced a bias which PMM has reduced. Alternatively, it is possible that passive imputation is giving less biased estimates than JAV and the complete-case analysis, but this seems unlikely in view of the simulation results presented earlier. It is perhaps surprising that the passive approach has yielded a larger estimated quadratic effect than the complete-case analysis; in the simulations the converse was true. This shows that the conclusions from our simulation studies may not apply when there is heteroskedasticity.

The SEs when using MI are somewhat smaller than those from the complete-case analysis, indicating that MI has made use of information from the subjects with missing data. This gain in efficiency is greater for JAV than for PMM. Had there been more missing values in the confounders, we would have expected a greater efficiency gain from using MI.

## Discussion

In this article, we have investigated imputation of an incomplete variable when the model of interest includes as covariates more than one function of that variable. We have focused on linear regression with a quadratic or interaction term, and have examined three imputation methods that can be easily implemented in standard software. In STATA, for example, the ice command can be used for passive imputation and PMM, and the mi impute mvn command for JAV; in R the mice function can be used for passive imputation and PMM, and the mix library for JAV. Note that although ice and mice use chained equations and hence, in general, involve iteration, when the data are monotone missing, as is the case in our simulation studies, no iteration is required.

In the JAV approach, each function of the incomplete variable is treated as an unrelated variable and a multivariate normal imputation model is used. Von Hippel (2009) claimed that this would give consistent estimation for linear regression when the data were MAR. In this paper we have shown that the consistency actually requires MCAR; when data are MAR, bias is to be expected. None of the three MI methods we investigated worked well in all the MAR scenarios considered. In general, JAV performed better than passive imputation or PMM for linear regression with a quadratic or interaction effect. We have shown, however, that there are circumstances in which JAV can have large bias for the quadratic effect of a linear regression model. JAV was found to perform very badly when the analysis model is a logistic regression, unless the outcome is common and covariates only have small effects on its probability. In view of this, we recommend that, given the current state of available software, JAV is the best of a set of imperfect imputation methods for linear regression with a quadratic or interaction effect, but should not be used for logistic regression. For logistic regression, the best performing imputation method was PMM. However, when *X *(and *X*^2^) are the only covariates in the model and are MAR, the complete-case analysis is unbiased, and hence we recommend its use in that case.

In our simulations, we found that using PMM was nearly always better than using passive imputation without PMM. However, for linear regression analysis models, its performance was usually worse than JAV.

In the scenarios we considered in our simulations, the analysis model only involves one variable (*X*) and its square, or two variables (*X *and *Z*) and their interaction. We have not considered additional covariates. We limited our investigation to these simple cases, because it is important to understand the performance of the methods in these scenarios before moving on to more complicated scenarios. Further research is needed into the performance of the methods when additional covariates are involved. In the scenarios we considered, the complete-case analysis is unbiased when the data are MCAR and is actually more efficient than all of the imputation methods considered. However, MI will be more efficient than just using complete cases when the analysis model involves additional non-fully observed covariates, because individuals with observed *X *and *Y *but missing other covariates will be excluded from the complete-case analysis but do contribute information when MI is used. Furthermore, the complete-case analysis may be biased when the data are not MCAR.

We (like Von Hippel, 2009) have presented JAV as a method using a multivariate normal imputation model. If the data are MCAR, JAV with this imputation model will give consistent point estimation in linear regression. The principle of JAV, i.e. that functions of the same variable are treated as separate and the functional relation between them ignored, is not tied to the normal distribution. However, the properties of a method using the JAV principle with another imputation model are thus far unknown.

## Conclusions

JAV gives consistent estimation for linear regression with a quadratic or interaction term when data are MCAR, but may be biased when data are MAR. The bias of JAV can be severe when used for logistic regression. JAV is the best of a set of imperfect methods for linear regression with a quadratic or interaction effect, but should not be used for logistic regression.

## Competing interests

The authors declare that they have no competing interests.

## Authors' contributions

IRW proposed the study. All authors made substantial contributions to the direction of the study, the design of the simulation studies, the interpretation of the results, and the writing of the manuscript. SRS carried out the simulations and data analysis and drafted the manuscript. All authors have read and approved the final manuscript.

## Pre-publication history

The pre-publication history for this paper can be accessed here:

http://www.biomedcentral.com/1471-2288/12/46/prepub
